# Bacteriological Quality of Table Eggs in Moroccan Formal and Informal Sector

**DOI:** 10.1155/2022/6223404

**Published:** 2022-09-24

**Authors:** Fatima Zahra El Ftouhy, Saâdia Nassik, Sabrine Nacer, Ahlam Kadiri, Nadia Charrat, Kawtar Attrassi, Asma Fagrach, Mohammed Amine Bahir, Sophia Derqaoui, Abdelaziz Hmyene

**Affiliations:** ^1^Laboratory of Biochemistry, Environment and Agri-food, Faculty of Science and Technology Mohammedia, University Hassan II, Casablanca, Morocco; ^2^Avian Pathology Unit, Department of Veterinary Pathology and Public Health, Hassan II Agronomic and Veterinary Institute, Rabat, Morocco; ^3^Laboratory of Bioscience, Exploration, Integrated Functional and Molecular FST, Mohammedia, Morocco; ^4^Microbiology Immunology and Contagious Diseases Unit, Department of Veterinary Pathology and Public Health, Hassan II Agronomic and Veterinary Institute, Rabat, Morocco; ^5^Department of Food and Environmental Microbiology of the Royal Gendarmery, Rabat, Morocco; ^6^Laboratory of Microbiology and Molecular Biology, Faculty of Sciences, Mohammed V University, Rabat, Morocco

## Abstract

Eggs constitute an important part of the Moroccan diet. However, contaminated eggs can cause a serious public health problem if consumed undercooked, uncooked, or used in unpasteurized egg foodstuffs. This study was carried out to evaluate the microbial contents of eggs according to their sales sector in Morocco. For that, a total of 1770 eggs were collected from January to September 2021 from formal markets (refrigerated eggs from large shopping centers) and informal markets (eggs at ambient temperature from ambulatory sellers, street vendors, kiosks, and neighborhood stores) and transferred to the Avian Pathology Unit at Hassan II Agronomic and Veterinary Institute. The eggshells and their contents were tested separately; swabs of eggshells were used to inoculate Mac-Conkey agar, while the egg contents were cultured on Mac-Conkey and Mannitol salt agar, then standard microbiological tests were performed to identify the isolated organisms. The results showed that informal eggs were more contaminated (87%) than formal eggs (48) (*p* < 0.05). The bacteria isolated from the eggshells (informal and formal) were *Enterobacter agglomerans* (59% and 21%), *Klebsiella* spp. (24% and 4%), *Enterobacter cloacae* (17% and 8%), *E. coli* (9% and 1%), *Serratia* spp. (9% and1%), *Pseudomonas aeruginosa* (9% and 1%), *Shigella* spp. (5% and 0%), *Salmonella enteritidis* (0% and 2%), *Proteus* spp. (4% and 0%), *Enterobacter sakazakii* (2% and 0%), *Rahnella aquatilis* (1% and 0%), and *Staphylococcus aureus* (0% and 1%). For the egg-contents, the detected bacteria (informal and formal) were *Enterobacter agglomerans* (14% and 28%), *Klebsiella* spp. (7% and 6%), *Staphylococcus aureus* (6% and 1%), *Enterobacter cloacae* (4% and 4%), *E. coli* (4%, 1%), *Shigella* spp. (4%, 0%), *Acinetobacter baumannii* (3% and 1%), *Salmonella enteritidis* (2% and 0%), *Serratia* spp. (1% and 6%), *Proteus* spp. (1% and 3%), and *Enterobacter sakazakii* (1% and 0%). We conclude that eggs might be contaminated with several bacteria and can constitute a public health threat in Morocco.

## 1. Introduction

Food safety is a major public health issue worldwide. Indeed, the prevalence of foodborne diseases remains extremely high in all countries [1]. Consumption of food contaminated with harmful microorganisms such as bacteria, viruses, and parasites are the main cause of this type of illness [2].

Among the most consumed foods is the egg, an important source of nutrients necessary for the development and vital functioning of organisms including humans. Its availability, low cost, and richness in high quality proteins, basic amino acids, vitamins, and minerals necessary for a good well-being [3], making it a major food in the human diet as well as attractive to consumers.

According to FISA (2020), the production of table eggs in Morocco has increased from 3.7 billion units in 2010 to 5.5 billion units in 2020. This production level allowed an average annual egg consumption in 2020 of about 177 eggs per capita.

The egg is a precious food not only by its nutrients necessary for the development of the living organism but also by its defense system to protect the embryo against microorganisms' infections. This defense mechanism is constituted by mechanical barriers (eggshell and shell membranes) and a biological barrier, mainly, different proteins of albumen with antimicrobial properties, in particular Lysozymes [4]. Similarly, the pH and viscosity of the albumen inhibit the proliferation of bacteria [5].

However, those various nutrients create a favorable environment for the multiplication of microorganisms, particularly, pathogenic bacteria such as *Staphylococcus aureus*, *Campylobacter jejuni*, *Escherichia coli*, and especially enterobacteria [6, 7]. These germs are responsible for serious foodborne diseases. The significance of these diseases can vary from mild symptoms to life threatening situations [8]. In addition, the microbial contamination of egg has an important outcome to the poultry industry.

The predominant sources of contamination can be classified as vertical and horizontal contaminations. Vertical contamination is the infection of the egg during its formation in the oviduct. It occurs when laying hens carry pathogens and transmit them to the egg. While horizontal contamination, which is more common, relates to the contamination of the surface of the eggshell after laying. It occurs by contact of the eggshell with a contaminated surface. In fact, feces, water, caging and nesting materials, insects, hands, broken eggs, dust on egg belts, blood, and soil are the most common sources of eggshell contamination [6, 7]. Changes that occur during egg packaging and storage can also facilitate the contamination of eggs by affecting their defense system. These changes can cause thinning of the albumen and the deterioration of the vitelline membrane and chalazas.

Despite all the efforts made throughout the production process of the egg, from farm to fork, the risk of consumer contamination is omnipresent.

The objective of the current study is to evaluate the importance of the bacterial contamination of eggshells and table egg contents according to their sales' sector: formal and informal, in Morocco.

## 2. Material and Methods

### 2.1. Sample Collection

A total of 1770 eggs resulting from 590 samples were collected during the period from January to September 2021 from different cities in Morocco: Kenitra, Sale, Rabat, Temara, Mohammedia, Casablanca, and Benslimane.

Eggs were collected from 2 different sectors: formal, mainly supermarkets in large shopping centers that sell refrigerated eggs, and informal from ambulatory sellers, street vendors, kiosks, and neighborhood markets selling eggs mainly exclusively at ambient temperature.

Clean eggs (with no visible flaws on the shell or cracks) were collected in sterile plastic bags and transferred aseptically to the microbiology laboratory of Avian Pathology Unit at Hassan II Agronomy and Veterinary Medicine Institute in Rabat, Morocco.

### 2.2. Preparation of Samples for Microbiological Examination

Once in the laboratory, the eggshells and their contents were tested separately. For the eggshells contamination, the swab technique was applied. In fact, the surface of whole eggs was aseptically swabbed with a sterile cotton swab moistened in a sterile distilled water solution. Then the eggs were disinfected by soaking in 70% ethanol for 5 to 10 seconds and air dried next to the Bunsen burner before content egg analysis. Actually, eggs' contents were transferred and pooled in a sterile stomacher bag before finally blending them to get a homogeneous mixture.

Both swabs and a mixture of eggs' contents were used to inoculate appropriate enrichment media and broths. It is important to mention that 3 pooled eggshells were considered as one sample; similarly, their contents were pooled to form one sample.

### 2.3. Bacteriological Analysis

Swabs of eggshells were used to inoculate Mac-Conkey agar. The plates were incubated aerobically overnight at 37°C. Both lactose fermenting and nonlactose fermenting colonies were picked from Mac-Conkey plates and subcultured on blood agar to purify the colonies.

For the eggs' content, a loopful of inoculum was streaked onto Mac-Conkey and Mannitol salt agar. The inoculated plates were incubated at 37°C for 24 to 48 h. Isolated colonies for Mac-Conkey were treated the same way as mentioned previously.

For *Salmonella* isolation, the eggs content (1 ml) was transferred to water peptone buffer and incubated at 37°C for 18 to 24 h. Some (0.1 ml) of the preinoculated water peptone buffer was transferred to Rappaport-Vassiliadis Soja (RVS) and incubated at 42°C for 24 h. A loopful of RVS was transferred to XLD agar and incubated at 37°C for 24 to 48 h according to the Moroccan standard NM ISO 6579, 2007 (NM 08.0.103).

All the isolated bacteria were identified by their morphology, color, shape, and color change of culture media. They were also stained using gram stains and examined with light microscope X 100 using oil immersion. Furthermore, biochemical tests were performed including coagulase, catalase, oxidase, and motility. This detection was also confirmed by API 20E for further biochemical identification.

## 3. Statistical Analysis

The data obtained in this study were analyzed using the Statistical Package for the Social Sciences (SPSS) version 22. One-way analysis of variance (ANOVA) was used to determine the significant differences between contamination of formal and informal eggs; eggshell load and their contents for each bacterium found plus eggshell load and egg contents from the 2 main sectors (formal and informal) for each bacteria found as well. *P* < 0.05 was considered statistically significant.

## 4. Results

### 4.1. Formal and Informal Egg Analysis

**Figure 1:**
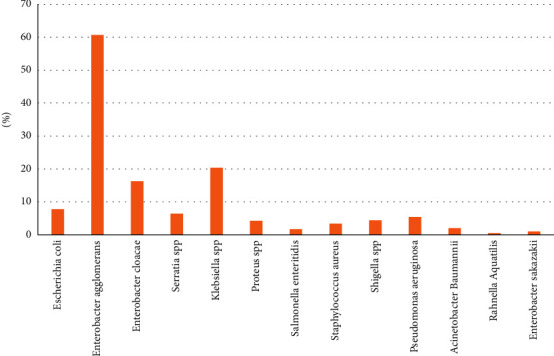
Distribution of bacterial organisms identified from all eggs' samples.

Between the formal and informal sectors, the highest percentage of bacterial contamination was noted in the informal sector with 87% of contamination, while the formal one revealed contamination of 48% (*P* < 0.05). In the tested eggs, the most common isolated bacteria were identified as belonging to the Enterobacteriaceae family. *Pseudomonas aeruginosa*, *Staphylococcus aureus*, and *Acinetobacter Baumannii* were the only non-Enterobacteriaceae members identified, and *Staphylococcus aureus* was the only gram-positive bacteria. The predominantly identified Enterobacteriaceae from samples were *Enterobacter agglomerans* (61%), *Klebsiella* spp. (20%), *Enterobacter cloacae* (16%), *E. coli* (8%), *Serratia* spp. (6%), *Shigella* spp. (4%), *Proteus* spp. (4%), *Salmonella enteritidis* (2%), *Enterobacter sakazakii*, and *Rahnella aquatilis* (1%). In addition, 5% of the samples were positive to *Pseudomonas aeruginosa*, 3% and 2% were *Staphylococcus aureus*, and *Acinetobacter baumannii* positive, respectively (Figure 1).

**Figure 2:**
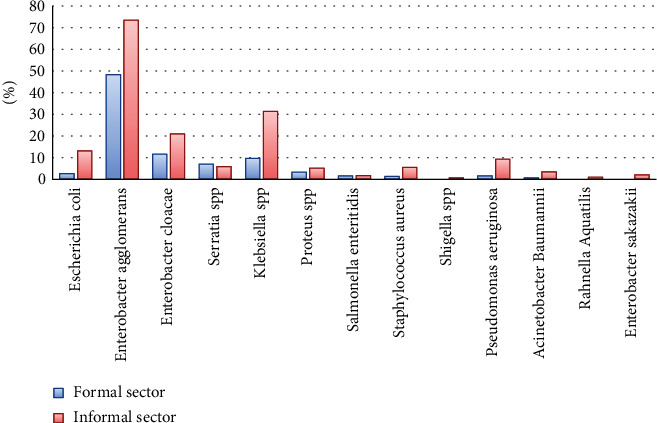
Bacterial organisms identified from eggs of the formal and informal sector.

As shown in Figure 2, the difference between the formal and informal sectors on bacterial load depended on the bacterium. Indeed, the prevalence of bacterial species isolated from the informal sector eggs were *Enterobacter agglomerans (*73%), *Klebsiella* spp. (31%), *Enterobacter cloacae* (21%), *E. coli (*13%), *Shigella* spp. (9%), *Pseudomonas aeruginosa* (9%), *Serratia* spp. (6%), *Staphylococcus aureus* (6%), *Proteus* spp. (5%), *Acinetobacter baumannii* (3%), *Salmonella enteritidis* (2%), *Enterobacter sakazakii (2%)*, and *Rahnella aquatilis (1%).* In the other hand, the bacteria species isolated from the formal eggs were *Enterobacter agglomerans*, *Enterobacter cloacae*, *Klebsiella* spp., *Serratia* spp., *E. coli*, *Proteus* spp., *Pseudomonas aeruginosa, Salmonella enteritidis*, *Acinetobacter baumannii*, and *Staphylococcus aureus* with a percentage of 48%, 12%,10%, 7%, 3%, 3%, 2%,2%, 1%, and 1%, respectively (Figure 2).

### 4.2. Formal and Informal Eggshell Analysis

**Figure 3:**
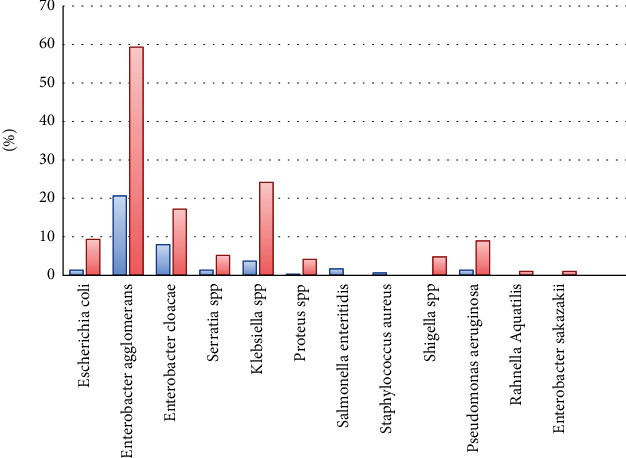
Bacterial organisms identified from shell surfaces of formal and informal eggs.

The obtained results show that a significant difference between the bacterial contamination of eggshells is noticed in the formal and informal sectors (*P* < 0.05). Bacteria isolated from the informal sector eggshells were *Enterobacter agglomerans* (59%), *Klebsiella* spp. (24%), *Enterobacter cloacae* (17%), *E. coli* (9%), *Serratia* spp. (9%), *Pseudomonas aeruginosa* (9%), *Shigella* spp. (5%), *Proteus* spp. (4%), *Enterobacter sakazakii* (2%), and *Rahnella aquatilis* (1%); while *Enterobacter agglomerans* (21%), *Klebsiella* spp. (4%), *Enterobacter cloacae* (8%), *Salmonella enteritidis* (2%), *E. coli* (1%), *Serratia* spp. (1%), *Pseudomonas aeruginosa* (1%), and *Staphylococcus aureus* (1%) were isolated from the formal eggshells (figure 3).

### 4.3. Formal and Informal Egg-Content Analysis

**Figure 4:**
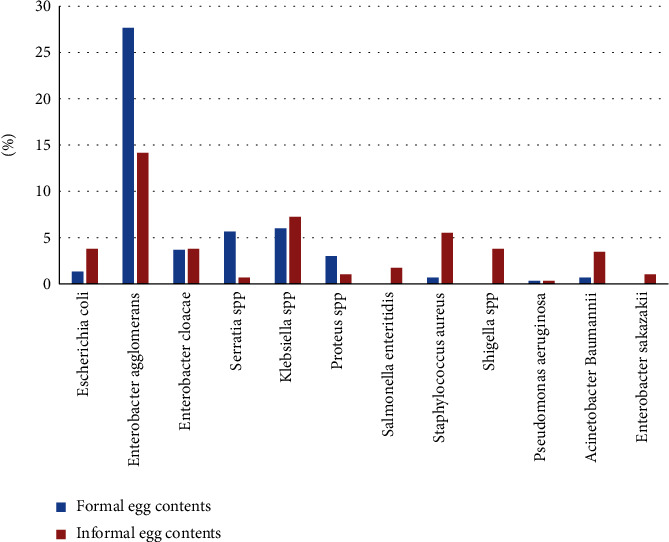
Bacterial organisms isolated from contents of formal and informal eggs.

The highest percentage of bacterial contamination on egg-contents was noted in the informal sectors (*P* < 0.05) that included *Enterobacter agglomerans*, *Klebsiella* spp., *Staphylococcus aureus, Enterobacter cloacae*, *E. coli*, *Shigella spp.*, *Acinetobacter baumannii*, *Salmonella enteritidis*, *Serratia* spp., *Proteus* spp., and *Enterobacter sakazakii* with a variable rate. In the meanwhile, the pure colonies isolated in the formal egg contents were identified as *Enterobacter agglomerans* (28%), *Klebsiella* spp. (6%), *Serratia* spp. (6%), *Enterobacter cloacae* (4%), *Proteus* spp. (3%), *Staphylococcus aureus* (1%), *E. coli* (1%), and *Acinetobacter baumannii* (1%). *Salmonella enteritidis* and *Shigella* spp. were not detected in the formal egg content; in addition, the results revealed the absence of pseudomonas in both egg contents of the two sectors (Figure 4).

## 5. Discussion

With the increased consumption of eggs and egg products, it is required to investigate the contamination of eggs to avoid any potential public health problems.

The main contaminants in all the samples were Gram-negative bacteria of the Enterobacteriaceae family, although *Staphylococcus aureus*, *Pseudomonas aeruginosa*, and *Acinetobacter baumannii* were also isolated at a lower level, as reported earlier [9, 10]. This result suggests that during the laying process, the egg goes through the common portion of the reproductive and digestive tracts where contamination can occur. The existence of a pathogen in the hen's ovary or oviduct before the shell is formed may end into of microorganism's presence in an intact or unbroken egg. Furthermore, the longer the eggs are left, the more their resistance decreases, allowing fecal contaminants to penetrate the egg contents.

### 5.1. Eggshells Quality in the Formal and Informal Sector

In this study, among the microbes isolated from eggshells of both sectors*, Enterobacter agglomerans* was the dominant with a high percentage in the informal markets. As previously reported, the bacteria that most frequently isolated from eggshells are Gram-negative bacteria such as *Enterobacter* spp. [11, 12]. This outcome could be due to the soil and/or water or to poultry workers handling eggs.

Among the shell's pathogenic bacteria in informal eggs, there were *Klebsiella* spp., *E. coli*, *Pseudomonas aeruginosa*, *Shigella* spp., and *Enterobacter sakazakii*, and for those in the formal sector, there were *Klebsiella* spp., *Salmonella enteritidis*, *Staphylococcus aureus, Pseudomonas aeruginosa*, and *E. coli*. Actually, in Nigeria, *Klebsiella* spp., *E. coli*, and *Shigella* spp. were detected in eggshells from retail outlets (informal sector) [13]. The same groups of bacteria, in addition to *Pseudomonas aeruginosa*, were isolated from shells of informal eggs in Grenada, India [9]. In addition, Nigerian eggs analysis from supermarkets (formal sector) has revealed the presence of *E. coli*, *Salmonella* spp., and *Staphylococcus aureus*; in contrast to our study, *Shigella* spp. was also detected [14]. Moreover, *Pseudomonas aeruginosa* and *Klebsiella* spp. were isolated from eggshells of Iranian markets [15]. Hence, many factors could be involved in the contamination of eggshells by this variety of bacteria. The presence of *Shigella* spp. in the informal sector only may result from the humid and hot bedding, unsuitable conditions in livestock buildings, litter material, contaminated egg crates [16], poor hygienic handling, transporting, and commercialization. Whereas, the occurrence of *Salmonella enteritidis* and *Staphylococcus aureus* in the formal sector may be related mainly to the transshell contamination if the environment is heavily contaminated with dust, soil, feces, also the tolerance of Gram's positive bacteria to dry and harsh conditions [17].

### 5.2. Egg-Contents Quality in Formal and Informal Sector

Unlike eggshells, *Enterobacter agglomerans* contamination was strongly detected in the contents of formal eggs only. These results agree with other research that confirms the high presence of *Enterobacter* spp., including *Enterobacter agglomerans*, in eggs purchased from markets rather than farms [18]. This could be due to poor storage conditions, poor hygienic measures during production and handling, or the sloppy cleaning process of eggs prior to marketing that could allow bacteria to penetrate through the eggshell pores to the inner surface of the shells and then the contents.

After bacteriological examination of the contents of eggs from informal markets, the results revealed the isolation of five pathogenic bacterial species identified as follows: *Klebsiella* spp., *Staphylococcus aureus*, *Shigella* spp., *E. coli*, and *Salmonella enteritidis*. Whereas, the contents of formal eggs were contaminated with only three types of bacteria, namely, *Klebsiella* spp., *Staphylococcus aureus*, and *E. coli*. Our findings are supported by previous studies that showed the presence of *E. coli*, *Klebsiella* spp., *Shigella* spp., *Salmonella* spp., and *Staphylococcus aureus* in the contents of eggs purchased from informal markets [13, 19], or *Salmonella* spp. and *E. coli* [20, 21]. On the other hand, in South Africa, *E. coli*, *Klebsiella* spp., and also, *Salmonella* spp. were isolated from the yolk and albumen of eggs from the formal sector [22]. In addition, as previously reported in Bangladesh, formal egg contents were contaminated with *E. coli*, *Staphylococcus aureus*, and *Salmonella* spp. [23]. During laying, eggs are sterile due to natural physical and chemical defense systems against microbial infection. However, this high bacterial contamination recorded in the egg contents of all samples examined can be attributed to the fact that the laying hens carried the pathogen prior to eggshell formation. In addition, dirt in the nest can contaminate the eggshell, and therefore, the bacteria can translocate from the outside to the inside of the egg.

Furthermore, the presence of *Salmonella enteritidis* and *Shigella* spp. in informal egg contents and not in the formals one may be related to the lack of adequate refrigeration as well as good storage facilities, which contributes to a rapid decline in egg quality.

Isolated bacteria have the potential to cause major health problems imposing important clinical and epidemiological challenges. In fact, about 80% of human *Salmonella* infections caused by serovars *Enteritidis*, *Heidelberg*, and *Hadar* in 1998–2008 period were attributed to eggs or poultry, with *Salmonella enteritidis* causing the majority of outbreaks [24]. This pathogen is responsible for foodborne human gastroenteritis, a disease characterized by gut inflammation and self-limiting diarrhea [25]. A study carried out prior to the implementation of egg safety rules by the Food and Drug Administration in 2010 reported that between 1973 and 2009, a total of 1,328 outbreaks of *Salmonella enteritidis* were recorded by the Centers for Disease Control and Prevention, resulting 40,767 estimated illnesses, 4,325 hospitalizations, and 104 deaths [26]. Most of these outbreaks have been attributed to eggs or egg products.

*Escherichia coli* can bring about urinary tract infections, pneumonia meningitis, and peritonitis in humans [27]. In fact, in northern Germany, from May to July 2011, a large number of hemorrhagic diarrhea cases were recorded. The shigatoxin-producing *Escherichia coli* strain, serotype O104:H4 (STEC O104:H4), triggered an outbreak involving approximately 4000 patients, including more than 900 with the hemolytic uremic syndrome (HUS), and 55 deaths [28].

Concerning *Klebsiella* spp., it can cause a wide range of community-acquired and nosocomial infections, such as urinary tract infections (UTIs), respiratory tract infections, and infections of wounds and soft tissue [29]. Infections caused by carbapenem-resistant *Klebsiella pneumoniae* (CRKP), for example, have gradually been reported all over the world [30]. In the Indian subcontinent, CRKP has become a major endemic [31]. Moreover, the European authorities have reported local outbreaks in various countries across the continent, with a high rate of CRKP in bloodstream isolates in Greece, Italy, and Cyprus [32].

For *Shigella* spp., diarrhea is an early symptom of its infection called shigellosis and may be initiated as the bacteria reach the small intestine. However, the bacteria predominantly target the colonic epithelium that they rapidly invade, causing inflammatory colitis [33]. This bacterium is considered the second-leading cause of diarrheal deaths after rotavirus, responsible for roughly 164300 annual deaths worldwide (12.5% of all diarrheal deaths), including 54900 in children under 5 years of age [34].

Unlike the mentioned bacteria, *Enterobacter sakazakii* may cause a highly lethal syndrome of bacteremia and meningitis in neonates [35], and a risk to immunocompromised adults, particularly the elderly. In 2007, mortality due to infections from *Enterobacter sakazakii* was higher than 50% [36]. In the same year, the pediatric unit of a hospital in Bilbao, Spain reported an *Enterobacter sakazakii* infection where a premature and underweight infant had contracted neonatal sepsis [37].

In the other hand, *Pseudomonas aeruginosa* is considered the most common pathogen responsible for both acute respiratory infections in ventilated or immunocompromised patients and chronic respiratory infections in cystic fibrosis patients [38, 39]. At the University of Pittsburgh, *Pseudomonas aeruginosa* was the most frequent cause (20%) of 670 cases of ventilator-associated pneumonia (VAP) [40]. This severe complication is related to considerable crude mortality of 42.1% to 87% [41] and high attributable mortality of 32% to 42.8% [41–43]. *Staphylococcus aureus* species can cause various infections such as food poisoning, endocarditis, osteomyelitis, skin infections, and pneumonia [44]. Indeed, *Staphylococcus aureus* is a significant cause of foodborne diseases, causing an estimated 241,000 illnesses per year in the United States [45]. Interestingly, a study involving 7126 cases indicated that case fatality rate of *Staphylococcal* foodborne disease is 0.03%; all deaths were in elderly patients [46].

The threat of these bacteria is limited not only to the illnesses they can cause but also to their antibiotic resistance in case of conventional antimicrobial treatments. This resistance is increasing worldwide over time despite the efforts and strategies adopted to confront it. Taking the profile of *Salmonella* spp. as an example where numerous studies have indicated an increase in the isolation of quinolone-resistant strains of Salmonella spp in several countries such as Germany [47, 48], England, and Wales [49]. Other studies detected increases in resistance to ampicillin and to nalidixic acid in the *Salmonella enteritidis* isolated from foods associated with salmonellosis occurring from 1999 to 2006 in the Rio Grande do Sul, Brazil [50].

The fact that egg might be defiled at each shell and contents by variety of organisms with a wide range of pathogens might reflect public health negatively in Morocco. Whereas, the risk of human contamination with contaminated eggshells could be much higher than with the contents of the eggs and can occur during storage or handling. Indeed, handling contaminated eggshells and the lack of hygiene of the operator leads to a high risk of contaminating other products, the culinary preparation during a possible manipulation, or to contaminate the operator himself. This behavior can contribute to the propagation of pathogens all over the working surface, which increases the risk of contaminating raw edible foods (fruits and vegetables). In some crops, chicken eggshell is a source of calcium (Ca), which is available at home that can be used as Ca supplementation [51]. This ancestral practice could also be the cause of a possible contamination if the shell is not well washed, dried, and disinfected. While a possible contamination by the egg content is only possible through consumption of insufficient cooked eggs and preparations made with raw eggs (mayonnaise, sauces, and chocolate mousse).

## 6. Conclusion

Eggs sold in Morocco regardless their sector sales might contain potential pathogenic bacteria that may cause a serious public health issue if handled, consumed undercooked, raw, or used unpasteurised in egg products. Thus, egg consumption should be considered with caution to avoid or at least reduce human foodborne illness. Preventive practices must be maintained before and after egg collection. In addition, the storage and marketing process must also respect and follow strict hygienic measures in order to prevent microbial growth on or in eggs.

## Data Availability

The authors declare that they have all the necessary data and are available where appropriate or requested by the editor.
